# Supplementary material for: Are carbon emissions trading and green financial instruments synergistic? -Comprehensive quantitative research based on content analysis
Source: PLoS One. 2024 Mar 7;19(3):e0298601. doi: 10.1371/journal.pone.0298601 (PMC10919601; doi:10.1371/journal.pone.0298601)
Supplement: S1 Data — (ZIP) [file pone.0298601.s001.zip › Data/地级市Z_F工作报告.zip!/╔╜╢1⁄2/┴─│╟╩╨/└┤╫╘╣1⁄2╓┌║┼í╛┬φ┐╦╩2╛▌═°í┐_2018.docx]

各位代表：

　　现在，我代表市人民政府向大会报告工作，请予审议，并请市政协各位委员提出意见。

　　一、2017年工作回顾

　　刚刚过去的2017年，是聊城发展历程中极不平凡的一年。一年来，全市上下坚持以习近平新时代中国特色社会主义思想为指导，深入贯彻党的十八大、十八届历次全会和十九大精神，按照省委、省政府和市委的部署要求，突出抓好“1+5”重点工作，全市经济社会保持持续健康发展。预计全市生产总值完成3100亿元，比上年增长7.5%；地方一般公共预算收入186.5亿元，同口径增长2% (扣除不可比因素,增长12.9%)；社会消费品零售总额增长9%；城乡居民人均可支配收入分别增长8%和8.5%，居民消费价格控制在1.5%。

　　工作中，重点抓了以下六个方面：

　　（一）着力推进转型升级，经济发展质量明显提升。认真践行新发展理念,全面推进供给侧结构性改革，下大气力优化产业结构、提升产业层次，预计三次产业比例调整为11.6：49：39.4，税收占财政收入比重提高4.5个百分点。农业结构持续优化。农业产业化、组织化、科技化、品牌化进一步加快，粮食生产实现“十五连丰”，优质小麦良种基本实现全覆盖；全市新改建高效集约蔬菜温室7.1万个，新增规模以上龙头企业30家、农民专业合作社1317家、家庭农场221家；发展节水灌溉面积42万亩，农作物耕种收综合机械化水平达到88%；开通了京沪高铁“聊·胜一筹！”专列，优质农产品品牌进一步打响，平均每天直供京沪蔬菜50多万斤。工业经济提档升级。“强链、延链、建链”扎实推进，全市铜加工能力达到90万吨、铝加工能力达到220万吨，鲁西集团精细化工和化工新材料产品占比达到80%以上，服装、家纺、产业用等三大类纺织品占到纺织行业的27%，高新技术产业产值占规模以上工业比重提高1.5个百分点。预计100家重点企业主营业务收入增长10%左右，100个重点项目完成投资340亿元。全市规模以上工业利税、利润均增长6%。服务业扩量提质。预计服务业增加值增长10%，占生产总值比重提高1.2个百分点。组建了市旅发集团，京杭运河保护开发样板段开工建设，阿胶小镇成功获批国家级特色小镇，全市旅游消费总额增长13.5%。全市建成电商产业园区8个、淘宝村级服务站300个，实现网络交易额1100亿元，冠县获批全省电子商务示范县。质量强市成效显著。主导或参与制修订各类标准12项。在全省率先建成质量大数据中心，建成国家铜铝产品质检中心和省级钢管、棉纱产品质检中心。全市达到省级农产品质量安全市标准，莘县等6个县（市、区）通过省级农产品质量安全县验收，东阿、高唐获批国家级出口食品农产品质量安全示范区。全市新增中国驰名商标1件、地理标志商标5件、山东名牌18个、山东老字号2个、“三品一标”186个。东阿阿胶荣获中国质量奖提名奖、国际QC金奖，中通客车荣获省长质量奖。

　　（二）着力激发动力活力，经济发展动能明显增强。以改革、开放、创新为动力，以项目建设为支撑，推动新兴动能加快形成、传统动能改造提升。深化改革力度加大。组建了注册资本10.2亿元的市级担保公司，成立了规模100亿元的城市发展股权投资基金和60亿元的产业发展基金。推进营改增试点，落实各项减税降费政策，为企业减负11.95亿元。农村集体产权制度改革试点稳步推进，承包土地经营权抵押贷款试点有序展开。完成规范化企业改制245家，新引进金融机构7家，新增新三板挂牌企业6家，全市直接融资184亿元。市县级公立医院、科研机构完成法人治理结构改革。出台了贯彻省支持非公有制经济发展十条意见实施方案，建立了市场公平竞争审查制度，全市新登记市场主体7.2万户，私营企业达到8.1万户，分别增长29%和32%。扩大开放力度加大。预计进出口总额增长16%，实际利用外资增长59.1%，分别居全省第6位和第2位。对接京津冀、长三角、珠三角成效显著，签订合同项目138个，计划投资1653亿元；预计全市招商引资到位资金800亿元。授予6位国内外人士“荣誉市民”称号。科技创新力度加大。全市新增省级以上创新平台15家、创新孵化平台7家。设立了1亿元的人才创新发展基金，引进外国专家104人、国内高层次人才40人、泰山产业领军人才5人。优质高效投入力度加大。预计固定资产投资增长10%，100个市级大项目完成投资582亿元。莘南高速、青兰高速建设加快推进，高唐至东阿高速开工建设，济郑高铁、京九高铁（雄安-聊城-商丘段）、聊泰铁路、聊城机场等前期工作均取得重大进展，国省道干线改造升级完成134公里。

　　（三）着力加强城乡建设，新型城镇化水平明显提高。全市完成住房和城乡建设投资390亿元，8.6万农业转移人口和6.1万城中村、城边村原有居民实现市民化。规划引领进一步强化。聊城市城市总体规划正式获批。编制了聊茌东都市区空间发展战略规划。中心城区完成了历史文化街区、绿地系统、综合交通等16项专项规划，修编了历史文化名城保护规划。各县（市）新一轮总体规划、乡村建设规划编制工作全面展开，完成乡（镇）总体规划78个。城市功能进一步完善。实施了“三河三湖”连通工程、东昌湖提升改造工程，开通了水上公交，望岳湖建设加快推进。建设了凤凰苑、九州洼、二干渠等城市生态公园，中心城区新增提升绿化面积314公顷。棚户区改造新开工3.5万套、基本建成2.8万套，公共租赁住房基本建成3836套、新增实物配租5829套，改造老旧小区121个。全市完成海绵城市建设23平方公里、地下综合管廊6公里、人防工程8.1万平方米。城市交通进一步改善。黑龙江路东延、茌东大道建成通车，湖南路西延主体完工，大外环建设拆迁工作基本完成。实施了城市交通“优化设计工程”，机动车通行效率提高11.5%。精细化管理进一步加强。城市违法建设治理扎实推进，拆除违建247万平方米；实行了城镇容貌和环境卫生管理考核制度，推进环境卫生网格化监管，市容市貌持续改善。农村环境进一步优化。创新实施“路长制”，路域环境明显提升；新改建农村公路3135公里、改造危桥140座。对24个小城镇进行了改造提升，新增79个乡镇（街道）通上天然气。美丽乡村建设扎实推进，1549个村居实现硬化路“户户通”，农村无害化卫生厕所基本实现全覆盖。

　　（四）着力抓好节能减排，生态环境质量明显好转。坚持以铁腕抓好生态环保，狠抓国家节能减排财政政策综合示范城市、国家循环经济示范城市建设，狠抓中央、省环保督察反馈问题整改，成功列入国家北方地区冬季清洁取暖试点城市、省级装配式建筑示范城市。全市淘汰小火电机组80.6万千瓦，清理取缔“地条钢”生产企业12家、“散乱污”企业3113家，关停电解铝违规产能53万吨，规模以上工业企业煤炭消费大幅度削减。全部取缔非法散煤经营，全面淘汰20蒸吨以下燃煤小锅炉，全市83台燃煤机组全部实现废气超低排放。大力实施清洁取暖工程，推广使用清洁能源9.8万户。全面推行“河长制”，“清河行动”进度居全省前列，提标改造污水处理厂18家。广泛开展植树造林，实施铁路沿线绿化186公里，新增森林、湿地公园3处，全市完成绿化面积25.2万亩。去年，我市主要河流COD改善4.9%、氨氮浓度改善51.8%，消除劣V类水体比例达到75%；可吸入颗粒物、细颗粒物、二氧化硫、二氧化氮浓度分别改善9.93%、17.4%、41.9%和2.44%，蓝天白云天数增加45天，是我市近年来空气质量改善幅度最大、效果最明显的一年。 经过全市上下共同努力，我们的天更蓝了、水更清了、地更绿了，人民的幸福感更强了！

　　（五）着力保障改善民生，人民群众生活明显改善。全市民生支出达到304.5亿元，占到财政总支出的80%。精准扶贫成效明显。全市发放金融扶贫贷款46亿元，建成扶贫养驴场100个，1015家企业带动贫困户2.9万人，邻里互助护理贫困群众2932人。“四大片区”建成项目321个、完成投资20.9亿元，金堤河扶贫大道、干流河道治理和桥梁改建全面完工，位山湿地公园正式运营。全市退出省定贫困村213个、脱贫7.87万人。社会保障更加有力。全市城镇登记失业率控制在3.04%；出台了职工大病保险和长期护理保险制度，居民医保财政补助、企业退休人员养老金、城乡低保等标准全面提高。教育事业加快发展。全市新建、改扩建幼儿园138处，聊城一中新校和聊城市特殊教育学校建成使用；普通中小学新增教学班2701个，城镇大班额问题基本解决；“全面改薄”开工率、竣工率均居全省前列，国家级义务教育均衡县实现全覆盖。文化体育事业更加繁荣。《铁血将军》等3部作品入选山东省“文艺精品工程”，北京保利剧院成功入驻聊城，放映公益电影6.2万场，行政村综合文化服务中心覆盖率达到80%。第七届全民健身运动会成功举办。卫生计生服务水平持续提升。市二院通过三级甲等综合医院评审，市县两级急危重症孕产妇、新生儿救治中心全面建成；“八项免费工程”惠及群众60余万人次，减免费用7000多万元。社会保持和谐稳定。圆满完成党的十九大安保维稳任务，受到省委、省政府表彰；“一村一警务助理”实现全覆盖。基层法律服务群众实现“一站通”和“一线通”。安全生产事故起数和死亡人数分别下降53.3%和19.4%。食品药品评价性抽检合格率达到96%以上，冠县成功创建省级食品安全先进县。文明城市创建取得重要成果，市民文明意识明显增强，在全省非全国文明城市测评中取得第一名。新闻出版、社会科学、民族宗教、台湾事务、气象地震、妇女儿童、青少年、老龄、对口支援等工作，都取得新的成绩。

　　（六）着力推动作风转变，政府自身建设明显加强。严格落实党建主体责任，认真组织学习党的十九大精神，扎实开展“两学一做”学习教育，切实把从严治党要求落到实处。认真落实“企业工作日”“金融工作日”“群众工作日”制度，实行了领导干部公开接访常态化，帮助解决问题1253件。提请市人大常委会审议地方性法规议案3件，制定政府规章3件；办理人大代表建议190件、政协委员提案206件，满意率和基本满意率分别达到98%和100%。制定了重大行政决策程序规定，法律顾问参与处理事项165件。大力推进多证合一，实行了“一窗受理、一站办结、一口出证”，市级“最多跑一次”许可事项占比达到51%，其中12项实现“零跑腿”。市长公开电话接通率和办结率均保持98%以上。开展了电视问政，改进了民主评议政风行风工作。各级审计为财政增收节支21.9亿元。纪检监察机关查处违反中央八项规定精神问题392起，查处群众身边不正之风和腐败问题1770起，给予党政纪处分1873人。

　　各位代表，2017年，党的十九大安保维稳任务重、节能减排的压力大、经济运行中的困难多。在这种形势下，我们能够取得这样的成绩，确实不易。这是中央、省委省政府和市委坚强领导的结果，是各级各方面大力支持的结果，是全市上下团结拼搏的结果。在此，我代表市人民政府，向全市人民，向人大代表、政协委员，向各民主党派、工商联、人民团体和各界朋友，向驻聊部队官兵和中央、省驻聊各单位，向所有关心支持聊城发展的海内外朋友，致以崇高的敬意和衷心的感谢！

　　各位代表，在看到成绩的同时，我们也清醒地认识到，政府工作还存在许多不足，也面临不少困难和挑战。主要是：发展不平衡不充分的一些突出问题尚未解决，经济结构不尽合理，发展的速度、质量和效益还不够高；新旧动能转换不够快，创新能力不够强，新经济的规模还比较小；城镇化水平仍然较低，城乡管理和公共服务还不完善；环境污染问题仍较突出，民生保障水平仍然较低，社会文明程度还不够高；政府职能转变还不到位，部分干部能力素质和担当精神不够强，不正之风和腐败问题时有发生。对此，我们一定高度重视，着力加以解决。

　　二、2018年的目标任务

　　2018年是贯彻党的十九大精神的开局之年，是改革开放40周年，是决胜全面建成小康社会、实施“十三五”规划承上启下的关键一年，也是聊城撤地设市20周年。今年政府工作的总体要求是：全面贯彻党的十九大精神，以习近平新时代中国特色社会主义思想为指导，坚持稳中求进工作总基调，突出新发展理念，紧扣社会主要矛盾变化，按照高质量发展的要求，统筹推进“五位一体”总体布局和协调推进“四个全面”战略布局，以供给侧结构性改革为主线，以新旧动能转换重大工程为统领，全面做好稳增长、促改革、调结构、惠民生、防风险各项工作，推动质量变革、效率变革、动力变革，确保高质量发展迈出坚实步伐、三大攻坚战取得扎实进展、社会事业全面发展、社会大局持续和谐稳定。

　　综合考虑多方面因素，建议全市经济社会发展主要目标为：生产总值增长7.5%左右，地方一般公共预算收入增长5%左右，固定资产投资增长10%左右，社会消费品零售总额增长9.5%左右，进出口总额保持稳定增长，城乡居民人均可支配收入分别增长8%和8.5%左右，居民消费价格涨幅控制在3%左右；全面完成省下达的各类约束性指标。工作中，突出抓重点、补短板、强弱项，集中力量抓好以下八个方面：

　　（一）聚焦聚力供给侧结构性改革,确保实体经济发展实现新突破。坚持以“四新”促“四化”，紧紧围绕建立现代化经济体系，着力增强实体经济的质量优势。

　　推进工业经济转型升级。深入落实《〈中国制造2025〉聊城实施纲要》和八大产业转型升级实施方案，加大“强链、延链、建链”工作力度，促进工业经济向全球价值链中高端迈进。一是推进传统产业高端化。加大企业技术改造力度，利用三年时间，对全市规模以上工业企业实施新一轮高水平技术改造，推动企业应用新技术、创造新产品、融合新业态、催生新产业，打造发展新优势。支持铜铝深加工、化工新材料、品牌服装、新能源汽车配套等产业发展，努力提高高端产品、高品质产品比重。坚决淘汰落后产能，抓好“僵尸企业”处置。二是推进新兴产业规模化。围绕新能源、新材料、生物医药、节能环保、高端装备制造等产业，以聚碳酸脂、动力电池、晶体材料等产品为重点，加大支持力度，尽快实现规模化生产，高新技术产业产值占规模以上工业比重提高1.5个百分点。三是推进重点产业集群化。大力推动汽车及机械装备、有色金属及加工、金属板材、轴承、钢管等产业集群式发展，强化龙头企业带动、中小企业配套、科技平台支撑、专业服务保障，打造一批千亿级、五百亿级优势产业集群。四是推进产业发展融合化。推动互联网、大数据、人工智能与制造业深度融合，抓好中通客车、东阿阿胶国家级智能制造专项建设，支持企业开展两化融合体系贯标，积极培育智能工厂、网络协同制造、个性化定制等新模式新业态。五是推进要素投入集约化。集聚财税、金融、土地、人才等政策，大力实施“双百工程”，着力培育一批具有创新能力的领军企业。以“提质、扩容、增效”为重点，加强信发高新材料产业园、祥光生态工业园、鲁西化工新材料产业园、中通和时风新能源汽车产业园等重点园区建设，确保主要指标增幅高于全市2个百分点以上。

　　加快现代服务业发展。实施服务业“双50”工程，抓好50个优质项目建设、50户优势企业培育，确保服务业占生产总值比重提高1个百分点以上。一是大力推动文化旅游业发展壮大。全面实施京杭运河聊城段保护开发，争取聊城成为全国大运河文化带区域性中心城市，临清、阳谷成为重要节点城市，打造集运河文化、湖河景观、文化体验、休闲养生、户外运动于一体的精品文化旅游产业带。加快江北水城旅游度假区发展，严格规划实施，抓好望岳湖工程的开发建设，加大旅游、休闲、养生项目招商和建设力度，打造高质量旅游度假示范区。抓好景阳冈旅游区、洲际假日酒店等70个重点项目建设，重点营销水城水浒休闲等8条旅游线路，争取东昌湖、东阿阿胶创建国家5A级景区。实施乡村旅游提档升级工程，重点抓好30个示范项目建设。二是大力推动商贸物流业转型升级。加快中心城区商业网点规划修编，抓好万达欢乐小镇等重点商贸项目建设，推进农贸市场改造提升。实施大型专业市场创新转型工程，规划建设大宗商品铁路物流园区，推广“无车承运人”等新模式，促进城乡、区域、产业链之间物流服务均衡智能发展，打造区域性物流枢纽城市。以创建国家电子商务示范城市为抓手，抓好电商园区、电商企业培育，推动电商企业在农村建设仓储物流和服务中心，力争电子商务交易额增长15%以上。三是大力推动健康服务业培育成长。实施“健康+”工程，推动医疗、养老、体育、旅游等融合发展，构建大健康产业体系。鼓励有条件的医疗机构向养老、康复、养生等服务延伸，支持社会力量开办医养结合机构，重点抓好聊城医养中心、东阿中医药健康产业园等项目建设，打造医养结合示范市。全面抓好服务业发展，突出发展与先进制造业相配套的金融服务、科技研发等生产性服务业，积极发展信息消费、文化休闲等新兴服务业，促进服务业提速提质提效。

　　（二）聚焦聚力乡村振兴，确保农业农村发展开创新局面。坚持农业农村优先发展，按照“产业兴旺、生态宜居、乡风文明、治理有效、生活富裕”的总要求，大力推进农业农村现代化，加快由农业大市向农业强市转变。一是切实加快现代农业发展。以“三区”同建为抓手，把保障粮食安全作为农业现代化的首要任务，加快构建现代农业产业体系、生产体系、经营体系，不断提高农业创新力、竞争力和全要素生产率。着力推进农业产业化，培育壮大农业“新六产”，重点抓好产业融合项目30个，新增规模以上龙头企业30家。着力推进农业组织化，引导农民开展土地入股、土地托管，规范提升440家农民专业合作社示范社，鼓励发展家庭农场。着力推进农业科技化，完成11处高标准示范园区、10万个高效集约温室建设任务，培育科技示范主体3000个，打造2-3家具有全国影响力的“育繁推一体化”现代种业集团。着力推进农业品牌化，健全农产品质量安全监管体系、检测体系和可追溯体系，组建县级以农药、化肥为主的农业投入品集中配送中心，新增“三品一标”基地面积17万亩，争创国家级农产品质量安全市；完善“聊·胜一筹！”品牌运营和传播体系，深入实施“净菜进京入沪”工程，把我市打造成为京津冀和上海等大城市精致农产品供应地。二是切实抓好农业基础设施建设。加快建设现代化灌区，实施彭楼灌区改扩建、位山等灌区渠首闸改建和节水改造，加快完善农田灌排体系。健全农业社会化服务体系，推进主要农作物生产全程机械化。三是切实深化农业农村改革。健全推进城乡融合发展的体制机制，设立5000万元的奖补资金、3亿元的引导基金，鼓励引导工商资本参与农村振兴。落实农村土地承包关系稳定并长久不变政策，衔接落实好第二轮土地承包到期后再延长30年的政策。完善承包地“三权分置”制度、农民闲置宅基地和闲置农房政策，优化土地资源配置。深化农村集体产权制度改革，推进农村集体资产确权到户和股份合作制改革。实施村级集体经济发展三年行动计划，加大资金投入，盘活集体资产，发展特色产业，力争2020年基本消除集体经济空壳村。四是切实优化农村人居环境。加快建设生产美、生态美、生活美的美丽乡村，开展农村人居环境三年整治行动，大力改善路水电气暖讯房等基础设施，巩固城乡环卫一体化成果；抓好30个省级示范村、35个市级示范片区建设，2020年70%以上的村庄达到省级美丽乡村标准。五是切实加强农村基层基础工作。创新农村社会治理，健全自治、法治、德治相结合的乡村治理体系。扎实做好村“两委”换届工作。全面建立职业农民制度，加强懂农业、爱农村、爱农民的“三农”队伍建设，培训农民20万人次。各位代表，聊城是农业大市，乡村振兴任务艰巨、关系重大。我们要以更大的决心、更明确的目标、更有力的举措，推动农业全面升级、农村全面进步、农民全面发展，奋力谱写新时代乡村振兴新篇章！

　　（三）聚焦聚力优质投入，确保新旧动能转换增添新动力。紧紧围绕新旧动能转换，坚定不移地实施大项目带动战略，以优质投入促进产业提升。一是以更大力度开展精准招商。把招商引资作为推动高质量发展的战略举措，力争过亿元招商项目到位资金300亿元以上。强化产业招商，按照管产业必须管招商的原则，分产业制定招商规划，精准开展招商活动。强化专业招商，加强专业队伍建设，鼓励采取购买服务的方式开展委托招商。强化重点区域招商，深化与京津冀、长三角、珠三角等地大型企业、高校院所的联系，推进更广领域、更深层次的对接与合作。强化园区招商，加强与国内外相关产业集聚区的对接，努力在打造特色上实现突破。强化招大引强，有组织、有步骤地走访对接央企、中国500强、民营企业500强，对好项目实行一业一策、一企一策。二是以更大力度抓好重点项目建设。实施新旧动能转换重点项目100个，进一步完善协调推进、考核奖惩等机制，加快项目建设进度，确保实现“三个百分之百”。按照“新、好、大、实”的要求，以新一代信息技术、高端装备、现代高效农业、精品旅游等“十强产业”为重点，加强高水平项目库建设，争取更多项目列入全省规划。三是以更大力度推进重大交通项目建设。今年，要确保莘南高速建成通车，聊泰铁路黄河大桥、东阿至东平黄河公路大桥开工建设，力争济郑高铁、京九高铁（雄安-聊城-商丘段）开工建设，聊泰铁路、聊邯长高铁、聊城机场等取得突破性进展。2019年、2020年，青兰高速、高唐至东阿高速分别建成通车。四是以更大力度强化项目保障。对新旧动能转换重点项目，要整合优惠政策，调动和集中各方面的力量，全力予以推进。要优化审批流程，设立综合受理窗口，开通便捷绿色通道，力争45个工作日完成施工许可。要千方百计破解土地、资金等难题，大力推进土地一级开发、城乡建设用地增减挂钩等挖潜改造，实行“指标跟着好项目走”。要切实解决好征地拆迁、施工环境等问题，确保工程顺利实施。各位代表，推进供给侧结构性改革、加快新旧动能转换，项目建设是支撑。我们一定要以最佳的环境、最优的服务、最快的速度，推动更多好项目大项目在聊城落地生根、开花结果！

　　（四）聚焦聚力创新驱动，确保提升发展质量取得新成效。坚持世界眼光、国际标准、聊城优势，深入实施创新驱动、质量强市战略，为建设现代化经济体系提供战略支撑。一是突出科技创新。实施龙头企业核心竞争力塑造工程，新增省级以上技术创新平台14家以上。积极引导中小微企业走“专精特新”的路子，打造“隐形冠军”“小巨人”企业。大力培育高新技术企业，加强高新技术创业服务中心建设，积极吸引大学生创业企业入驻众创空间，引进科技型创业企业20家。全力支持高新区发展，突出高标准定位，推进智能装备、生物医药、环保科技等六大产业园建设，创建国家级高新区。二是突出质量引领。实施“标准化+”行动，主导或参与制修订各级标准8项以上，采用国际标准、国外先进标准5项以上；深化质量提升行动，整体提升鲁西质检中心水平，加快国家轴承产品质检中心、省中压输配电设备检测中心建设，推进冠县金属板材、开发区钢管等创建省级质量提升示范区；大力推进品牌建设，新注册商标4000件以上，争创山东名牌10个以上，培育1-2家企业申报山东省省长质量奖，支持阳谷特种光电线缆、临清轴承创建全国知名品牌示范区，全面完成全国质量强市示范城市创建任务。三是突出人才支撑。加大高层次人才培养力度，健全产学研用协同用人机制，支持企业与高校院所联合培养人才，实施好企业家培育“123”计划。加大高层次人才引进力度，实行更加积极的“高精尖缺”人才引进政策，与聊城大学共建大学科技园，确保引得进、留得住、用得好。加大高技能人才培养力度，加强职业院校建设，鼓励校企联合培养人才，做好劳动模范、水城工匠、首席技师选拔工作，让广大技能型人才有实实在在的成就感、获得感，努力造就一支知识型、技能型、创新型劳动者大军。各位代表，功以才成，业由才广。我们要实行更加积极、更加开放、更加有效的人才政策，不唯地域引进人才，不求所有开发人才，不拘一格用好人才，努力让各类人才在聊城大地发挥聪明才智、迸发创造活力！

　　（五）聚焦聚力改革开放，确保经济发展活力得到新提升。实施新旧动能转换，深化改革、扩大开放是动力和保障。扎实推进重点领域改革攻坚。以完善产权制度和要素市场化配置为重点，着力构建市场机制有效、微观主体有活力、宏观调控有度的经济体制，为高质量发展提供制度保障。深化国企国资改革，推进市属经营性资产统一监管，加快国有企业公司制、股份制改造，推进投资主体多元化。深化企业改革，全面推进个转企 、小升规、规改股 、股上市 ，突出抓好规模以上企业规范化公司制改革。 深化投融资体制改革，推进融资平台公司转型升级，鼓励县（市、区）设立产业发展基金、城市发展基金、直投基金，加大政府与社会资本合作工作力度。 深化金融体制改革，鼓励企业对接多层次资本市场，全市实现2-3家企业境内外上市，力争每个县（市、区）在创业板、中小板或新三板挂牌上市1家，全市直接融资130亿元以上；培育壮大地方金融机构，推动普惠金融发展，不断改善中小企业金融服务。 深化商事制度改革，抓好重点领域价格改革，推进行业性协会商会和行政机关脱钩，打破行政性垄断。支持民营经济发展，鼓励更多社会主体投身创新创业，全年新登记市场主体5万户以上，力争新增规模以上中小企业100家，培育省级“一企一技术”研发中心和创新企业10家以上。大力弘扬企业家精神，尊重企业家、关心企业家、保护企业家，积极引导企业家新老交替，注重新生代企业家培养，建立重点企业“直通车”制度，让企业家在聊城放心干、放手干，营造激励企业家干事创业的良好氛围！

　　全方位扩大对外开放。实施更加积极主动的开放战略，深度融入“一带一路”建设，推动全市新一轮高水平对外开放。一是更深层次融入区域发展战略。全面融入京津冀协同发展，推进产业发展对接协作、资源要素双向流动、生态环境共建共享、基础设施互联互通，打造“一区一枢纽五基地”。积极融入中原经济区、山东西部经济隆起带、省会城市群经济圈，深化与济南的融合对接。二是更大规模扩大利用外资。突出骨干企业利用外资，引进资金、技术、管理经验，提升国际化水平。突出产业链、价值链中高端利用外资，支持外资投向战略性新兴产业、高端制造业和现代服务业。突出招大引强，创新模式和方式，以更大的力度实施产业、专业招商，努力引进世界500强及全球行业领军企业。三是更高水平拓展对外贸易。优化进出口结构，加强国际自主品牌培育，努力扩大品牌商品出口。支持企业参加国际展会，设立境外销售网络，建立稳定的产品销售渠道。实施“互联网+外贸”企业培训计划，积极培育贸易新业态新模式，发展跨境电子商务。积极申报聊城保税物流中心，推进临清“两国双园”项目和海关监管场站建设。四是更大步伐建设“海外聊城”。加强国际产能合作，推动纺织、机械、造纸等传统产业建立海外生产基地，实现生产能力向海外延伸。推动骨干企业走出去，投资并购境外研发、设计、营销、品牌等价值链优质资源。深化境外能源资源合作开发，加强铝土矿、铜矿等基地建设。五是更大力度加快开发区发展。积极推动开发区体制机制创新，完善土地利用机制，提高招商引资质量，着力打造一批富有特色的“园中园”“区中园”，推动各级开发区转型升级、创新发展。

　　（六）聚焦聚力城乡建设，确保新型城镇化迈出新步伐。抢抓建设国家新型城镇化综合试点城市、全省大城市试点的机遇，加快推进以人为核心的新型城镇化，实现11万农业转移人口市民化和9万城中村、城边村原有居民市民化。一是着力提升规划水平。推进多规合一，加强中心城区规划编制，全面完成各县（市）新一轮总体规划、乡镇总体规划编制，实现城乡规划全覆盖。坚持刚性约束，维护规划的严肃性，坚决制止、严肃查处随意变更规划、违规乱建等行为。二是着力提升建设水平。加快全域水城建设，完成东昌湖景区提升工程，实施西南岸改造，策划建设大型灯光秀项目；抓好运河五期项目建设，全面推进二干渠、青年渠等中心城区主要水系及两岸生态修复提升，实施徒骇河、马颊河滨河道路、两岸绿化及引蓄水工程。加快聊茌东都市区建设，推进茌平、东阿撤县设区，构建“一带、两轴、三组团”发展格局。加快中心城区扩容提质，高水平规划建设高铁新城；抓好大外环建设，新建改造鲁化路、陈口路等47条道路，改造延伸主干道，着力打通断头路；开工建设综合管廊8公里，建成海绵城市6平方公里；大力实施森林进城围城工程，外环以内新增绿化面积2.5万亩，实现城市生态环境大幅提升。三是着力提升管理水平。推进城市管理市场化，引入竞争机制，实行“管办分离” “管养分离”。推进城市管理智慧化，建设智慧城市运行平台。推进城市管理精细化，完善网格化管理机制，加强便民市场和早夜市规范管理，确保市容市貌明显提升。四是着力提升县（市）城区和小城镇建设水平。积极推动临清建设中等城市试点，阳谷、高唐建设I型小城市试点。加强小城镇建设，加快陈集、烟店、博平、石佛等国家和省级特色小镇发展。

　　（七）聚焦聚力短板弱项，确保社会和谐稳定展现新面貌。着眼发展中不平衡、不协调的问题，切实打好三大攻坚战。全力打好防范化解重大风险攻坚战。一是切实抓好安全生产。深入开展“安全生产责任落实年”活动，加强重点行业领域专项整治，扎实推进化工产业安全生产转型升级专项行动，坚决防止重特大安全事故发生。二是切实维护社会稳定。深化平安聊城建设，完善网格化服务管理，强力推进“雪亮工程”，做好重大活动安保工作，推动基层基础工作提档升级。认真做好信访工作，及时回应群众合理诉求，妥善化解各类矛盾。三是切实防控金融风险。健全金融监管体系，强化属地风险处置主体责任，严控化解担保圈风险，守住不发生系统性金融风险底线。鼓励企业通过上市挂牌、私募基金、资产证券化、债转股等方式，有效降低负债水平。支持金融机构发挥主体作用，多措并举化解不良贷款。积极搭建资金供需双方有效对接平台，灵活运用财政资金，发挥 “四两拨千斤”的作用。多措并举整治金融乱象，坚决打击恶意逃废金融债务、非法集资等违法行为，推进互联网金融风险排查，做到早发现、早预警、早处置；支持金融监管部门加强行业监管，查处乱加杠杆、违规套利、盲目抽贷压贷等行为，努力维护好金融秩序和社会和谐稳定。

　　全力打好精准脱贫攻坚战。坚持精准扶贫精准脱贫基本方略，把提高脱贫质量放在首位，全面发力，精准施策，确保基本完成脱贫攻坚任务。一是加大片区攻坚力度。突出抓好金堤河、沉沙池、黄河故道等“四大片区”脱贫攻坚，加快基础设施、产业发展、民生工程项目实施，推动片区加快改变面貌。二是加大产业扶贫力度。深入推进养驴扶贫、光伏扶贫、电商旅游扶贫、金融扶贫，抓好十大扶贫项目建设，扶持33个省旅游扶贫村发展。扩大产业帮扶项目覆盖面，切实做到“一户一策”。三是加大行业社会扶贫力度。深入推进教育扶贫、健康扶贫、就业扶贫，建设扶贫车间50个以上，提升爱心众筹平台运营水平，引导更多企业、社会组织、爱心人士参与扶贫。四是加大长效机制建设力度。加强利益联结机制建设，建立完善项目运营管理机制、扶贫资产保值增值机制，使贫困群众长期受益。完善特困群体保障机制，推进集中供养，实现贫困户邻里互助护理全覆盖。把扶贫和扶志、扶智相结合，加强就业技能培训，激发贫困人口内生动力。

　　全力打好污染防治攻坚战。牢固树立绿水青山就是金山银山的理念，统筹山水林田湖草系统治理，全面建成国家节能减排财政政策综合示范城市。一是继续深化大气污染防治。坚持标本兼治、长短结合，深化供给侧结构性改革，发展绿色产业，努力减少能耗和污染物排放。深入抓好减煤工作，推进30万千瓦以下燃煤机组淘汰；全面取缔散煤经营，采用先进的节能环保取暖设备，推广清洁取暖20万户；全面推行建筑节能，完成太阳能光热建筑一体化80万平方米以上，中心城区新开工装配式建筑占比不低于20%、县（市）不低于12%。深入抓好扬尘治理，认真落实“路长制”，抓好路域环境整治；全面落实建筑工地“六个百分之百”，抓好煤炭、渣土、砂石料等运输车辆管理，引导大宗货物通过铁路运输。深入抓好清洁生产，强化重型柴油车管控，严控挥发性有机物污染。二是全面抓好水污染防治。全面落实“河长制”，持续开展“清河行动”，坚决查处“八乱”现象。加强污水处理厂提标建设，重点河流两岸乡镇污水处理厂全部建成。利用三年时间，全面完成污水收集管网“雨污分流”改造。严格执行养殖区域规划，引导建设规范化养殖厂房，防止养殖业污染。全面推进水资源保护、水污染防治、水环境治理、水生态修复，清除黑臭水体，维护河湖健康生命。三是有序推进土壤污染防治。开展土壤污染状况调查，加强土壤环境监测，提升危废综合处理能力，抓好工矿废弃地复垦利用。四是大规模开展城乡绿化。深入实施“六大工程”，加快8个国有林场改造提升，抓好森林乡镇、森林村庄建设，新增绿化面积15万亩以上，确保成功创建国家森林城市。各位代表，抓好环境保护，必须坚定信心、保持定力，夯实责任、动真碰硬。今年，我们要进一步强化执法监管、强化考核奖惩，用最严格的制度、最严密的法治，推动全市环境质量得到更大改善，把我们的家园建设得更加美好！

　　（八）聚焦聚力社会民生，确保群众生活水平获得新提高。牢固树立以人民为中心的发展思想，加大保障改善民生力度，不断满足群众日益增长的美好生活需要。

　　全面发展各项社会事业。优先发展教育事业，落实立德树人根本任务，发展素质教育，努力减轻中小学生课外负担；推动城乡义务教育一体化发展，全面完成农村义务教育薄弱学校改造任务；深入实施中小学校长职级制和“县管校聘”教师管理体制改革，鼓励社会力量办学，支持发展民办教育。加强健康聊城建设，推进医联体建设和发展，巩固提升分级诊疗制度，推行以按病种付费为主的医保支付方式，鼓励支持社会办医；深入开展爱国卫生运动，做好全国卫生城市复审工作；加强计划生育管理服务，加快发展老龄事业。推动文化事业繁荣发展，加强文艺精品创作，传承发展黄河文化、运河文化、水浒文化、红色文化、民俗文化等聊城优秀传统文化，加快“一校三馆”建设，基本实现乡（镇、街道）综合文化站、村（社区）综合文化服务中心全覆盖。广泛开展全民健身运动，建设全民健身工程300处，举办第八届全民健身运动会，办好自行车、马拉松、龙舟等大型体育赛事。扎实做好双拥共建、革命老区工作，推进聊城革命烈士陵园迁建。加强精神文明建设，弘扬和践行社会主义核心价值观，深入推进“四德”工程建设，开展移风易俗、弘扬时代新风行动，争创全国文明城市。全面做好新闻出版、社会科学、气象地震、残疾人、人防、对口支援等工作，努力取得新的成绩。

　　扎实办好十件民生实事。一是促进就业创业。加大公益性岗位开发力度，完善聊城创业服务网络平台、公共招聘平台，做好困难群体就业工作，培训劳动者1.5万人以上，发放创业担保贷款3.6亿元以上，全市城镇新增就业4.5万人。二是改善群众居住条件。加大棚户区改造力度，新开工4.8万套、建成1.9万套，改造老旧小区104个；发展住房租赁市场，建成公共租赁住房1156套。三是建设幼儿园和中小学校。加强对现有幼儿园的规范管理，提升幼儿园办园水平；实施第三期学前教育三年行动计划，推行民办公助，新建、改扩建幼儿园100所。建设中小学34处，新增教学班520个，全面解决大班额问题。四是发展养老服务机构。新增医养结合养老机构4家，发展城市社区日间照料中心，新增养老床位2600张。五是提高保障标准。提高城乡低保保障标准、优抚对象抚恤补助标准，将建档立卡贫困家庭全部纳入医疗救助范围。六是改善农村道路条件。加强“四好农村路”建设，新改建农村公路800公里，改造危桥80座；农村“户户通”硬化路完成2600个村居、6800公里。七是实施中心城区交通“优化提升工程”。对城区道路进行交通优化设计，排查提升交通安全设施，加大“微循环”治理，主干道通行率提高15%以上。八是实现管道天然气乡镇全覆盖。建设武城-阳谷、冯官屯-高集等4条天然气主管道，新增供气能力9亿立方米，确保所有乡镇通上天然气。九是提高文化惠民水平。举办公益演出2000场，放映公益电影6万场，保利水城明珠剧院举办高水平惠民演出50场。十是严格食品药品监管。实施餐饮质量提升工程，莘县、东阿争创省级食品安全示范县。各位代表，群众的小事就是我们的大事。我们要时刻把群众的安危冷暖放在心上，一件接着一件办，一年接着一年干，努力让全市人民生活得更有尊严、更为便利、更加幸福！

　　三、加强政府自身建设

　　打铁必须自身硬。新时代、新任务必须要有新气象、新作为，全市各级政府一定要强化“四个意识”，推进现代政府治理体系和治理能力建设，推动政府工作再上新水平。

　　（一）加强能力建设，增强施政本领。按照建设高素质专业化干部队伍的要求，在全市政府机关营造善于学习、勇于实践的浓厚氛围，建设学习型政府。把政治建设摆在首位，深入学习党的十九大精神，扎实开展“不忘初心、牢记使命”主题教育，用习近平新时代中国特色社会主义思想武装头脑。加强专业知识学习，培养提高干部的专业能力、专业精神。大兴调查研究之风，善于发现经验，找准短板弱项，增强工作的预见性、针对性和实效性。坚持学以致用，做好科学理论、先进经验、聊城实际结合的文章，在学中干、干中学，切实增强“八个本领”，牢牢把握工作的主动权。

　　（二）加强效能建设，优化政务环境。围绕打造精简高效的政务生态，深化改革、优化流程、强化服务。扎实推进“放管服”改革，进一步取消和下放行政审批事项；推进“双随机、一公开”，构建各部门协同共治监管格局；加强“互联网+政务服务”建设，推进“多评合一”，实现市级“最多跑一次”许可事项占比达到70%以上。下决心清理涉企收费，严厉查处和整治乱收费，努力降低实体经济成本、制度性交易成本。加快推进机关事务集中统一管理，降低行政成本，提升服务水平。认真落实“企业工作日” “金融工作日” “群众工作日”制度，扎实做好政务公开、来信来访、建议征集、市长公开电话、民主评议政风行风等工作，构建“亲”“ 清”新型政商关系，提高群众对政府工作的满意度。

　　（三）加强法治建设，严格依法行政。严格执行市人大及其常委会的决议决定,自觉接受人大法律监督、工作监督和政协民主监督,主动听取民主党派、人民团体和社会各界意见建议,认真办理人大代表议案建议和政协委员提案。积极配合人大立法，促进立法和各项改革决策相衔接。加强与决策咨询委员会的沟通联系，严格执行咨询论证制度。支持共青团、妇联等群团组织更好地开展工作，认真落实党的民族宗教和侨务政策，做好新时期外事、对台工作。全面落实重大行政决策程序规定，完善合法性审查、风险评估、责任追究等制度，做到科学民主依法决策。深化行政执法体制改革,推动跨部门综合执法和执法力量向基层下沉，进一步提高政府治理能力和执法监管水平，让市场和社会既充满活力又规范有序。

　　（四）加强作风建设，倡树实干担当。九层之台，起于累土，我们必须不驰于空想、不鹜于虚声，一步一个脚印，踏踏实实地干好工作。严格执行中央、省委省政府和市委的各项重大决策部署，落实主体责任，确保令行禁止、政令畅通。强化领导带头，落实领导干部一线工作法，切实发挥示范引领作用。完善目标责任制管理，对重点工作制定任务书、责任单、时间表、流程图，切实做到可操作、可检查、可评估、可考核。加大督导检查力度，健全问责处罚机制，全面整治庸政、懒政、怠政行为。坚持激励和约束并重，完善考核评价体系，更加注重实绩考核，更多地引入第三方评估，严格兑现奖惩。抓好市委、市政府《关于激励干部担当作为干事创业的意见》的落实，大力弘扬担当精神，切实为担当作为者保驾护航、为勇于创新者撑腰鼓劲，营造干事创业的良好氛围。

　　（五）加强廉政建设，保持清正廉洁。坚持严字当头，牢牢把握“全面从严在路上”的过硬标尺，推动政府系统全面从严治党向纵深发展。加强财政预算绩效管理，严控“三公”经费，实现市县公共资源交易平台一体化。深化审计管理体制改革，创新审计方式，提升监督效能，强化问题整改，加强审计结果运用。认真落实中央八项规定实施细则精神和省委实施办法，大力整治形式主义、官僚主义，防止“四风”问题反弹回潮。深入推进反腐败斗争，强化不敢腐的震慑，扎牢不能腐的笼子，增强不想腐的自觉，保证干部清正、政府清廉、政治清明。

　　各位代表，空谈误国、实干兴邦，幸福都是奋斗出来的。让我们更加紧密地团结在以习近平同志为核心的党中央周围，深入贯彻习近平新时代中国特色社会主义思想，在省委、省政府和市委的坚强领导下，凝心聚力，埋头苦干，为决胜全面建成小康社会、实现聊城人民对美好生活的向往而努力奋斗！
